# Making the Final Cut — Mechanisms Mediating the Abscission Step of Cytokinesis

**DOI:** 10.1100/tsw.2010.129

**Published:** 2010-07-20

**Authors:** John A. Schiel, Rytis Prekeris

**Affiliations:** Department of Cell and Developmental Biology, School of Medicine, University of Colorado, Aurora, CO, USA

**Keywords:** cytokinesis, abscission, endosome, ESCRT, membrane traffic, secretory pathway

## Abstract

Cytokinesis is the final stage of mitotic cell division that results in a physical separation of two daughter cells. Cytokinesis begins in the early stages of anaphase after the positioning of the cleavage plane and after the chromosomes segregate. This involves the recruitment and assembly of an actomyosin contractile ring, which constricts the plasma membrane and compacts midzone microtubules to form an electron-dense region, termed the midbody, located within an intracellular bridge. The resolution of this intracellular bridge, known as abscission, is the last step in cytokinesis that separates the two daughter cells. While much research has been done to delineate the mechanisms mediating actomyosin ring formation and contraction, the machinery that is responsible for abscission remains largely unclear. Recent work from several laboratories has demonstrated that dramatic changes occur in cytoskeleton and endosome dynamics, and are a prerequisite for abscission. However, the mechanistic details that regulate the final plasma membrane fusion during abscission are only beginning to emerge and are the subject of considerable controversy. Here we review recent studies within this field and discuss the proposed models of cell abscission.

Our understanding of cytokinesis in mammalian cells has evolved from an event driven exclusively by an actomyosin contractile ring–mediated system, to a process that now incorporates membrane traffic, originally thought only to occur in plant cells. Cytokinesis is initiated from signals emanating from the mitotic spindle, which mediate the formation and contraction of an actomyosin ring, ingression of the cleavage furrow[1], and finally lead to the formation and resolution of a thin intracellular bridge that connects two daughter cells[2]. The process of furrow ingression is well understood and relies predominately on myosin II–dependent actin ring contraction[1]. In contrast, abscission appears to require cross-talk between multiple cellular pathways, which includes microtubule and actin cytoskeleton rearrangements, as well as endocytic and secretory organelle transport to the furrow[3]. Indeed, after completion of furrowing, the daughter cells remain connected by the intracellular bridge for up to a few hours, presumably to undergo extensive changes in membrane and cytoskeleton networks. Multiple studies, including analysis of midbody proteome[4], along with work on the endosomal sorting complex required for transport (ESCRT)[5,6,7,8,9], the Exocyst complex[10,11], Rab11 and Rab35 GTPases[10,12,13], secretory traffic[11], cytoskeleton[14,15,16], and lipids[17,18,19,20,21,22], have demonstrated that a series of complicated and highly orchestrated events must take place for the two daughter cells to undergo abscission. This mini-review will discuss the newest discoveries about the molecular machinery required for cell division and will discuss proposed models of cell abscission.

## THE INVOLVEMENT OF THE CYTOSKELETON DURING CYTOKINESIS

### Actin and Cell Abscission

Classically, cytokinesis was defined as occurring through the use of an actomyosin contractile ring composed of actin, nonmuscle myosin II, and septins[1]. Shortly after anaphase onset, the construction of an actomyosin ring begins at a predefined cleavage plane[14], followed by ring constriction and formation of a narrow intracellular bridge. Research in fission yeast has revealed that the contractile ring forms from myosin II– and actin-enriched nodes, which together with formin Cdc12p, serve to generate the force required to pinch the daughter cells in two[23]. The role for septins in cytokinesis is less clearly defined. In budding yeast, septin assembles into higher-order structures that serve to form a plasma membrane–associated lattice[24,25], which can recruit other contractile ring proteins, such as anillin[26]. The cross-linking protein anillin can bind all three filament types and acts as a scaffolding protein that can organize efficient actomyosin contractile ring assembly[27].

Contractile ring assembly is influenced by the Rho family of small GTPases that serve to regulate actin nucleation and myosin II activity[28]. In mammalian cells, members of this family include Rho (A, B, and C), Rac (1, 2, and 3), and Cdc42, and have been shown to be required for cytokinesis[29]. In animal cells, RhoA is critical in both contractile ring formation and the progression of the cleavage furrow during cytokinesis by promoting actin polymerization and myosin phosphorylation[30]. As with all small monomeric GTPases, Rho GTPases alternate between an inactive GDP-bound form and an active GTP-bound form. This activation/inactivation cycle is controlled by three classes of effector proteins: GTPase-activating proteins (GAP), guanine exchange factors (GEF), and guanine nucleotide dissociation inhibitors (GDI). To activate RhoA, a GEF is required to exchange its bound GDP for a GTP and during cytokinesis, this is accomplished by a RhoA-specific GEF, ECT2. ECT2 is recruited to the midzone of a dividing cell by binding to Cyk-4/MgcRacGap (hereafter Cyk-4) to coordinate RhoA activation and initiation of furrowing during cytokinesis[31,32]. Cyk-4 binds to MLKP1, forming a complex called centralspindlin, which is then recruited to the midzone of a dividing cell, important for the formation of the central spindle and actomyosin contractile ring[31,32]. Thus, RhoA activation and subsequent initiation of actomyosin complex contraction occurs through the coordinated efforts of centralspindlin and ECT2, and their recruitment to the midzone of a cell[29]. The role of Cyk-4 in cytokinesis and specificity for different Rho GTPase family members remains largely controversial. *In vitro*, the GAP domain of Cyk-4 has been shown to activate GTP hydrolysis of RhoA, Rac1, and Cdc42[33]. Unfortunately, full-length Cyk-4 has yet to be stably produced *in vitro*, contributing to the confusion surrounding the specificity of Cyk-4's GAP activity towards the different Rho family members. Two different models have been proposed for its activity towards the different Rho family members. One hypothesis, a Rac inactivation model, states that the GAP activity of Cyk-4 activates the GTPase function of Rac at the cleavage furrow, thus inactivating Rac and inhibiting branched actin nucleation by Arp 2/3, while promoting RhoA-dependent formation of linear actin filaments[34]. An opposing hypothesis, a Rho GTPase Flux model, postulates that Cyk-4 is a GAP for RhoA and by coordinating its activity with ECT2, establishes an activated RhoA zone at the cleavage furrow[35,36].

Once the actomyosin ring has fully constricted, the cell must undergo a process of dissembling the actomyosin ring to make way for abscission. Actin filament disassembly and turnover has been established during ingression of the cleavage furrow[37] and twinstar mutants, a *Drosophila* homolog of ADF-cofilin, display abnormally large cleavage furrows and defects in cytokinesis[38]. It is still unclear as to how exactly the actin cytoskeleton is regulated at late telophase, but its disassembly appears to be important for the abscission step of cytokinesis.

## Microtubules and Cell Abscission

The role of the microtubule cytoskeleton during cell division has been extensively studied and is well established. Indeed, during mitosis, different sets of centrosomal microtubules are required for the segregation of chromosomes (kinetochore microtubules), positioning of centrosomes (astral microtubules), and the formation of actomyosin contractile rings (astral microtubules). A different set of microtubules emanating from the centrosome are the interpolar or midzone microtubules that form the central spindle containing a bundled region of overlapping microtubules[39]. The concentration of the central spindle during furrow ingression will eventually give rise to a midbody, also known as Fleming's body. The central spindle and the midbody contain a variety of different microtubule-associated proteins with bundling activities, such as PRC1/Kif4 and the centralspindlin complex (composed of a kinesin-6 family member, MKLP1, and Rho GTPase family GAP,Cyk-4), which are required for central spindle and midbody formation[28,40,41]. During anaphase, the activation of the centralspindlin complex has been illustrated to alter the activation status of Rho/Rac family members and lead to the constriction of the actomyosin contractile ring[34,35,36]. Furthermore, the midbody is also involved in cell signaling, since it contains a variety of kinases that include aurora A, aurora B, and Plk1 kinases, all of which are known regulators of cytokinesis[42].

While the role of microtubules in mediating chromosome separation and furrow ingression is well understood, the function of microtubules during abscission remains to be determined. It is generally accepted that microtubules need to be removed from parts of the intracellular bridge in order to get final separation of the daughter cells. Consistent with that, abscission is usually initiated at the proximity of the midbody, presumably because the midbody itself prohibits plasma membrane fusion due to a highly dense and cross-linked microtubule network. The mechanisms that initiate the removal of microtubules at the site of the abscission still remain enigmatic. Two main models have been proposed recently. The first model suggests that microtubules may be severed during late telophase, thus establishing the abscission site. Consistent with that, microtubule-severing enzymes, such as katanin and spastin, have been demonstrated to be present at the intracellular bridge[43,44]. Furthermore, individual knock-downs of katanin or spastin appear to delay, but not completely block, abscission[43,44]. It also remains unclear how spastin or katanin are targeted and selectively activated at the parts of the intracellular bridge. An alternative model suggests that the microtubule network at the intracellular bridge may be regulated by minus-end disassembly, rather than microtubule severing. Unlike astral and kinetochore microtubules, the minus-ends of central spindle microtubules detach from centrosomes during metaphase/anaphase transition[45]. As a result, it was suggested that during anaphase and telophase, the length of the central spindle microtubules may be tightly regulated by minus-end binding proteins. Consistent with that, ASPM, the human homologue of the *Drosophila* Asp protein, is a microtubule minus-end binding protein that was asserted to be important in cytokinesis and central spindle stability in neuronal cells[46]. Additionally, TBCD protein (tubulin cofactor D) was shown to regulate midzone microtubule retraction at the midbody[47].

While microtubules need to be removed from the intracellular bridge just before abscission, the intact central spindle is required at the early stages of abscission. It has been shown that central spindle microtubules serve an important role in the delivery and accumulation of secretory organelles and recycling endosomes at the intracellular bridge[48]. Several kinesin molecular motors, namely Kinesin I and Kinesin II, were implicated in delivering endosomes to the furrow[49,50]. This plus-end–directed organelle movement is counterbalanced by minus-end motor dynein that was demonstrated to regulate endosome transport to the furrow during cytokinesis[51]. Thus, regulated switching between plus-end and minus-end motors seems to play a key role in regulating endosome delivery to the furrow, ultimately affecting the timing of the abscission step.

## DELIVERY OF VESICLES IS NECESSARY FOR CYTOKINESIS

In addition to the actin and microtubule cytoskeleton, membrane trafficking recently has emerged as an important player in mediating cytokinesis. Multiple, post-Golgi, membrane-bound organelles were revealed to accumulate at the intracellular bridge during late telophase[52]. Moreover, distinct changes occur in endocytic recycling during mitosis[53,54,55], such as the inhibition of membrane recycling to the plasma membrane during metaphase and anaphase, and a subsequent increase in membrane recycling during telophase. These temporal changes of membrane recycling seem to be required for successful completion of cytokinesis[53]. While the function of these furrow organelles remains unclear, organelle and plasma membrane fusion appears to be a key step in abscission as several SNARE proteins, such as syntaxin 2 and VAMP8, were established to be required for the late stages of cytokinesis in mammalian cells[11,56].

Work in several different organisms, including mammalian cells, *Caenorhabditis elegans*, and *Drosophila melanogaster* embryos, have shown that recycling endosomes play an important role in cell abscission. Consistent with this, Rab11, a small monomeric GTPase that is known to regulate recycling endosomes, has been delineated to be required for the late stage of cytokinesis[13]. Rab11 and its effector FIP3 are essential for the targeting of recycling endosomes to the cleavage furrow[10,13]. Knock-down of FIP3 via siRNA inhibits late cytokinesis, resulting in an increase in the number of bi- and multinucleate cells[13]. Furthermore, the *Drosophila* homologue of FIP3, nuclear fallout protein (Nuf), was found to regulate endosomal protein transport during cellularization of embryos[57]. The Rab11 and FIP3 protein complex functions by binding to Cyk-4 and the Exocyst complex, anchoring recycling endosomes at the intracellular bridge[10,58,59].

In addition to Rab11, Rab35 was also shown to be required for successful completion of cytokinesis. siRNA screen in *Drosophila* S2 cells was used to identify Rab35 as important for cytokinesis, and knock-down of Rab35 in S2 cells showed a multinucleate phenotype[60]. These results were confirmed in HeLa cells by knock-down and overexpression of a dominant-negative Rab35 mutant[60]. Additionally, it was found that endosomal recycling to the plasma membrane was impaired with Rab35 knock-down, leading to a decrease in furrow lipid PtdIns(4,5)P2 and septin component SEPT2[60].

Secretory vesicles have also been implicated in cytokinesis[11,61,62,63]. Using secretory GFP, it was demonstrated that after leaving the trans-Golgi network (TGN), secretory vesicles are transported to the intracellular bridge where they fuse with the furrow plasma membrane in a VAMP8-dependent manner[11,48]. The targeting of these vesicles to the furrow was demonstrated to be dependent on centriolin, a protein of maternal centrioles that can be found in the midbody during late telophase[11]. Centriolin depletion causes an increase in the prevalence of long intracellular bridges that failed to undergo abscission[11]. Interestingly, the recruitment of secretory vesicles by centriolin is also dependent on the Exocyst complex[11]. The Exocyst complex serves as a common anchor and tethering factor for both secretory vesicles and recycling endosomes[10]. Consistent with such a model, the knock-down of several components of the Exocyst complex inhibits cell progression through late telophase[10,11,58].

## LIPID DYNAMICS DURING CYTOKINESIS

In addition to proteins, phospholipids have emerged as important regulators of cytokinesis, although their role is much less understood. The existence of distinct lipid subdomains was reported within the intracellular bridge in mammalian cells. Characteristically, the inner leaflet of the lipid bilayer is enriched with phosphatidylserine (PS) and phosphatidylethanolamine (PE), while the outer leaflet is enriched with phosphatidylcholine (PC) and sphingomyelin[64]. Interestingly, it was demonstrated that, in dividing CHO cells, PE is exposed on the cleavage furrow plasma membrane surface during the final stages of cytokinesis[21]. If flipping of PE between inner and outer leaflets during cytokinesis is blocked by immobilization of PE, with a PE-binding peptide, cells exhibit strong cytokinetic defects[18]. Similarly, cytokinesis is also impaired in CHO cells deficient in PE[19].

Another phospholipid class, the phosphoinositides, has also been established to be required for cytokinesis[18,22]. Phosphoinositide 4-5 biphosphate (PtdIns(4,5)P2) is known to localize to the cleavage furrow of NIH3T3 fibroblasts, mouse macrophages, and HeLa and CHO cell lines. The accumulation of PtdIns(4,5)P2 at the furrow starts with the initial furrow ingression[22]. Furthermore, inhibition of PtdIns(4,5)P2 production by overexpressing kinase-dead phosphatidylinositol 4-phosphate 5-kinase mutants, or by microinjection of antibodies against PtdIns(4,5)P2, increase the multinucleate phenotype characteristic of failed cytokinesis[18,22]. Lastly, two enzymes, phosphatase and tension homologue on chromosome 10 (PTEN) and phosphoinositide 3-kinase (PI3K), which regulate the production of PtdIns(4,5)P2 and PtdIns(3,4,5)P3, have also been exhibited to be required for cytokinesis[65].

In addition to the requirement of phospholipids noted above, gangliosides also appear to be important for cytokinesis. By using *Xenopus laevis* eggs, it was shown that during anaphase, the ganglioside GM1 accumulates at the site of what is to become the intracellular bridge, and as the cleavage furrow ingresses, GM1 concentrates inside the bridge[66]. This enrichment requires actin and microtubules, since small molecule inhibitors of actin and microtubules disrupt GM1 and cholesterol enrichment at the cleavage furrow[66]. Additionally, in cholesterol-depleted cells, GM1 is unable to concentrate at the cleavage furrow[66]. Isolation of detergent-resistant membranes (DRM) containing GM1 and cholesterol revealed that Src and PLCγ are contained within these DRMs, that they are activated by tyrosine phosphorylation, and that this is important for cleavage furrow ingression[66].

The function of phospholipids during cytokinesis remains unclear. PE and PS were illustrated to regulate the disassembly of actin filaments and the actomyosin contractile ring[20,67,68]. Similarly, PtdIns(4,5)P2 binds and regulates many proteins that regulate actin polymerization[15]. Thus, PtdIns(4,5)P2 may be required for stabilization of the actomyosin ring during initial phases of cytokinesis. Finally, dynamic changes in PE and PtdIns(4,5)P2 levels may be responsible for mediating the disassembly of the actomyosin ring just before the abscission step of cytokinesis.

## ESCRT COMPLEX AND CYTOKINESIS

Relatively recent work on cell division has also implicated the ESCRT complex in mediating abscission[5,6,7,8,69]. The ESCRT complex was originally identified as a complex involved in the formation and fission of intraluminal vesicles during maturation of multivesicular bodies (MVB) (for review, see Gruenberg and Stenmark[70]). Later, it was revealed that the ESCRT complex is also required for outward budding of enveloped viruses[70,71]. The ESCRT complex consists of four protein complexes (ESCRT-0, -I, -II, -III) and can bind accessory proteins such as ALIX and VPS4, which aid in either recruitment of other ESCRT complexes or for ESCRT disassembly[72]. While ESCRT 0–II complexes are involved in the recruitment of cargo destined to enter intraluminal vesicles, the ESCRT-III complex was implicated in actual intraluminal vesicle formation and fission, presumably via oligomerization of Snf7 (mammalian CHMP4A/B)[73]. Indeed, Snf7, Vps24 (mammalian CHMP3), and Vps20 (mammalian CHMP6) are sufficient to cause vesicle scission *in vitro*[73]. CMHP2A and CHIMP3 were also shown to copolymerize and tubulate membranes *in vitro*[74].

Based on the ESCRT-III function in MVB formation and viral budding, it was suggested that the ESCRT complex also has a role in cytokinesis that is topologically equivalent to their respective roles in MVB biogenesis and viral budding[72]. Consistent with that, several ESCRT-III proteins have emerged to be required for cytokinesis[6,7,8,9,75]. For example, depletion of ALIX and Tsg101 inhibits abscission in HeLa cells and, in addition, point mutations within ALIX inhibit recruitment and binding of CHMP4 to CEP55 (centrosomal protein 55) at the midbody[8]. Furthermore, CHMP1B was shown to bind the microtubule-severing factor spastin, and mutations in CHMP1B and spastin binding domains impair cytokinesis[9,43]. While the exact mechanistic details of the ESCRT complex function during cytokinesis remain to be elucidated, good experimental evidence implicates the ESCRT complex in the regulation of the severing of furrow microtubules and/or resolving the intracellular bridge during cytokinesis.

## CURRENT MODELS FOR ABSCISSION OF DAUGHTER CELLS DURING CYTOKINESIS

In recent years, multiple studies have emerged demonstrating that cross-talk between membrane transport and cytoskeleton play a key role during abscission. With an ever-increasing number of proteins that affect the late telophase steps of cytokinesis, the challenge has become the incorporation of all the data into models explaining the machinery of cell abscission. At the moment, three competing, but not necessarily mutually exclusive, models for abscission have been presented: the compound fusion model, the ESCRT scission model, and the mechanical forces model (Fig. 1).

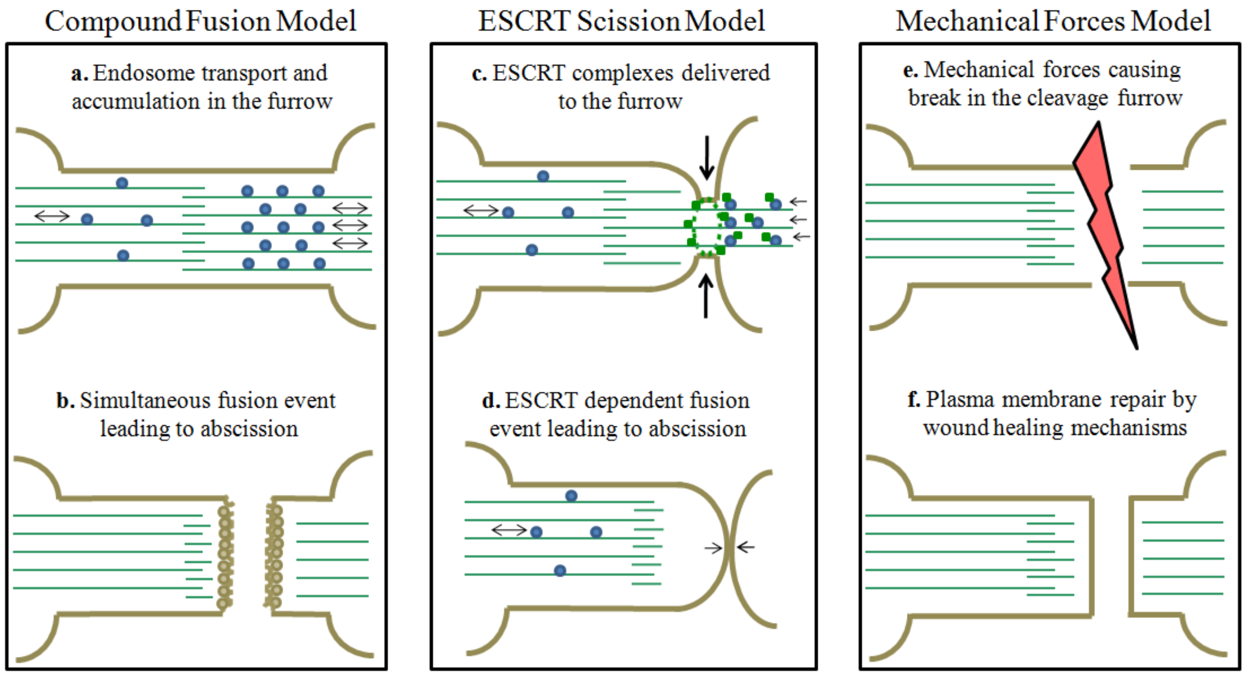

**FIGURE 1.** Models for cellular abscission. Brown, plasma membrane; light green, microtubules; blue, vesicles; dark green, proteins/lipids required for abscission. The top panels of each model (a,c,e) display earlier cytokinesis events, while the bottom panels (b,d,f) display late cytokinesis events. (a,b) Compound fusion model. (a) Post-Golgi and/or recycling endosomes are delivered and accumulate within the intracellular bridge. (b) Synchronous compound fusion leads to abscission. (c,d) ESCRT scission model. (c) Recycling endosomes are trafficked to the intracellular bridge delivering their membrane and cargo, possibly ESCRT proteins, leading to an ESCRT-dependent narrowing of the plasma membrane and disassembly of microtubules. (d) ESCRT-mediated scission event–based abscission. (e,f) Mechanical forces model of abscission. (e) Traction-based adhesion forces pull daughter cells apart, rupturing the intracellular bridge. (f) Plasma membrane repair through wound-healing mechanisms.

### Compound Fusion Model

The observation that recycling endosomes and secretory vesicles accumulate at the intracellular bridge at late telophase led to a hypothesis that simultaneous compound fusion of these organelles with each other and the furrow plasma membrane leads to the separation of daughter cells[11]. Here we refer to this as the compound fusion model (Fig. 1). The compound fusion model asserts that in early telophase, there is an orchestrated, asynchronous accumulation of post-Golgi organelles at the intracellular bridge adjacent to the midbody[11]. This accumulation is mediated by a secretory vesicle binding to centriolin via the Exocyst complex, as well as recycling endosome binding to the centralspindlin and the Exocyst complexes[10,11,59]. Eventually during late telophase, an unknown signal initiates mass compound fusion between organelles and the furrow plasma membrane, resulting in intracellular bridge resolution.

Consistent with this, it was demonstrated that the endocytic SNARE, VAMP8, and plasma membrane SNARE, Syntaxin 2, are required for abscission[56]. Furthermore, accumulation of post-Golgi organelles at the intracellular bridge are also required for abscission[48]. However, high-resolution analysis of furrow organelle accumulation and spatial distribution has not been done. Thus, due to the limitations of light microscopy, it is hard to tell whether organelles accumulate in the furrow in sufficient numbers to actually mediate such a compound fusion–dependent abscission. The synchronous fusion event just before abscission also remains to be demonstrated. In contrast, some evidence suggests that organelles continuously fuse with the furrow plasma membrane during telophase[48]. Finally, cytokinesis has been shown to be unaffected by treatment with Brefeldin A in the first two divisions of sea urchin eggs and in *Drosophila* S2 cells[62]. Thus, it is possible that endosomes may play some other roles during cytokinesis and do not directly mediate the abscission event. Perhaps recycling endosome trafficking is an earlier event that precedes abscission, possibly adding a membrane to each daughter cell at and near the intracellular bridge in order to re-establish its initial surface area[53] prior to abscission.

## ESCRT Scission Model

Recently, ESCRT proteins have emerged as potential mediators of cellular fission separating the two daughter cells. The ESCRT scission model is based on the premise that inward budding of MVB vesicles and outward viral budding are topologically similar to the events occurring in cytokinesis[75]. Since ESCRT proteins were indicated to be involved in viral budding and MVB formation, it was suggested that they may also mediate resolution of the intracellular bridge, presumably by forming a "collar" at the interior of the furrow plasma membrane and mediating the constriction of the intracellular bridge, sometimes referred to as secondary ingression. Consistent with that, the ESCRT-II protein Tsg101, the ESCRT accessory protein ALIX, and several ESCRT-III proteins have been found to localize to the midbody and were demonstrated to be required for cytokinesis[5,6,7,8,69]. Interestingly, CHMP1B was illustrated to bind the microtubule-severing enzyme spastin[9,43]. This observation provides an intriguing possibility that the ESCRT complex may coordinate microtubule clearance and intracellular bridge constriction. However, several major problems regarding the ESCRT scission model remain to be answered. It was shown that ESCRT proteins accumulate specifically at the midbody, yet abscission never occurs at the midbody. Usually, the intracellular bridge gets resolved on one side of the midbody, sometimes a considerable distance from the midbody. Thus, it is unclear how accumulation of ESCRT at the midbody can actually mediate the abscission. In addition, there are considerable topological differences between intraluminal vesicle budding and cytokinesis. ESCRT-mediated vesicle budding results in a remarkably homogeneous population of intraluminal vesicles that are between 24 and 50 nm in diameter[76]. Consistent with that, a CHMP lattice was shown to form tubules of about 45 nm in size[74]. In contrast, the intracellular bridge is about 1.5–2.0 μm in diameter, although in HeLa and D-98S (human sterna bone marrow) cells, the intracellular bridge was observed occasionally to narrow to about 0.2 μm in diameter[2,77]. Thus, the question remains, if and how can the ESCRT complex mediate scission of membrane tubes varying from 24 nm to 1.5 μm in diameter? Furthermore, the furrow plasma membrane has been demonstrated to be highly dynamic, exhibiting multiple plasma membrane "waves" during late telophase. Finally, while it was hypothesized that the ESCRT complex forms a "collar" on the furrow plasma membrane, it remains to be revealed whether midbody-associated ESCRT proteins are on the plasma membrane or on endocytic organelles, perhaps MVB/late endosomes. While the necessity of the ESCRT complex during cytokinesis was clearly demonstrated by many laboratories, the function of the ESCRT complex in cell abscission remains unclear. It is possible that the ESCRT complex may have other functions besides scission, such as degradation of the various proteins and protein complexes that must be removed for abscission to occur, and/or for the recruitment of other proteins, such as spastin, that are needed to sever microtubules in the intracellular bridge. Further investigation into this new area of ESCRT complex–mediated abscission is required.

## Mechanical Forces Model

The mechanical forces abscission model implies that by the use of physical force, cells undergo abscission and close the membrane tear at the site of abscission by wound-healing mechanisms[78]. Actomyosin-independent abscission was first identified in the slime mold *Dictyostelium discoideum* and was termed "Cytokinesis B" or "attachment-assisted mitotic cleavage"[79,80], and "Cytokinesis A" requiring the actomyosin contractile ring. In normal rat kidney (NRK) cells treated with the myosin II inhibitor blebbistatin, cytokinesis and abscission occur as long as these cells adhere to a substrate, which causes physical separation of the daughter cells due to cell motility[81]. When NRK cells were plated on agarose plates, on which the NRK cells could not adhere, cytokinesis was inhibited[81]. Furthermore, integrins and other cell-adhesion molecules were shown to be required for cytokinesis[82]. Thus, at least in some cell types, physical traction is required for completion of cell division. However, in the case of nonadherent cells, such as leukocytes and lymphocytes, these traction-based adherent forces will not be generated and will not contribute to cytokinesis and abscission. It is possible that other mechanical forces, besides adherent forces, may contribute to the abscission of mammalian cells. Proteomic analysis of mammalian midbodies identified the presence of the microtubule-binding motor dynein[4], which localizes to the plasma membrane of the intracellular bridge[83]. Perhaps by attaching itself on the plasma membrane, dynein can generate localized forces along microtubules that help drive abscission. Alternatively, filopodia pushing may contribute to abscission. Consistent with that, filopodia are highly enriched at the furrow ([2,84] and unpublished data), and it was demonstrated that a single filopodium can generate a 3 pN force[85]. Further studies will be needed to explore these possibilities.

## CONCLUSIONS AND FUTURE OBJECTIVES

Multiple studies during the last several years have considerably increased our understanding about cytokinesis and its terminal event, abscission. Although we have generated a component list of proteins participating in cytokinesis, it is still unclear how all of these proteins interact to drive cytokinesis and abscission. Additionally, functional redundancy and resolution limits of light microscopy have impeded progress. The abscission models reviewed here, while still incomplete and describing conflicting events, provide a good framework that we can improve upon. These conflicting models need not be mutually exclusive. It is likely that the mechanisms of abscission involve parts from all three models. Improved resolution in fluorescent live imaging methods, such as stimulated emission depletion microscopy (STED) and photo-activated localization microscopy (PALM), and the use of immuno-EM and high-resolution tomography techniques, will refine our spatial and temporal understanding of cytoskeleton and organelle spatial and temporal dynamics from furrow ingression to the late stages of intracellular bridge formation and scission. Advancements in ultrastructure analysis and in fluorescent light imaging techniques will hopefully be able to answer key questions plaguing these models, such as… how does the intracellular bridge resolve itself, what are the signals specifying abscission, and what mediates disassembly and/or severing of actin and microtubule cytoskeleton just before abscission?
